# Directional Response of Randomly Dispersed Carbon Nanotube Strain Sensors

**DOI:** 10.3390/s20102980

**Published:** 2020-05-24

**Authors:** Alfredo Güemes, Angel Renato Pozo Morales, Antonio Fernandez-Lopez, Xoan Xose F. Sanchez-Romate, Maria Sanchez, Alejandro Ureña

**Affiliations:** 1 Department of Aeronautics, Universidad Politecnica de Madrid, 28040 Madrid, Spain; angelorenato20@gmail.com (A.R.P.M.); antonio.fernandez.lopez@upm.es (A.F.-L.); 2 Area of Materials Science and Engineering, Universidad Rey Juan Carlos, 28933 Madrid, Spain; xoan.fernandez.sanchezromate@urjc.es (X.X.F.S.-R.); maria.sanchez@urjc.es (M.S.); alejandro.urena@urjc.es (A.U.)

**Keywords:** strain monitoring, bonded joints, multidirectional sensors, doped resins

## Abstract

Tests on a double lap bonded joint, with transverse strips of randomly oriented carbon nanotubes (CNT) sprayed onto an epoxy adhesive film, showed a positive increment in electrical resistance under tensile load, even though the transverse strains were negative. Other experiments included in this work involved placing longitudinal and transversal CNT sensors in a tensile loaded aluminum plate, and, as reported by other authors, the results confirm that the resistance change is not only dependent on the strains oriented with the electrode line, while the other strain components also influence the response. This behavior is quite different to that of conventional strain gages which have a near zero sensitivity to strains not aligned to the sensor direction. The dependence of the electrical response on all the strain components makes it quite difficult, possibly unfeasible, to experimentally determine the individual strain components with this kind of sensors; however, the manufacturing of aligned CNT sensors could deal with this issue.

## 1. Introduction

Continuum mechanics starts with the definition of the strain and stress tensors. The strain state at any point of an elastic solid is defined by the six independent components of the strain tensor. In most real cases (beams under flexural and torsion loads, thin plates and shells), simplified strain-stress fields are assumed, and many of the components of the strain and stress tensors are considered to be zero or negligible. However, even for the simplest case, a flat plate under uniaxial tensile load, three perpendicular strain components are still there, one extension aligned with the stresses and two lateral compressions, due to the Poisson effect. 

Experimental stress analysis [1] deals with methods and techniques that measure the strain components at the body surface, so the analytical or numerical models of the structure may be validated. Electrical strain gauges, used since 1938, are simply a metallic wire bonded to the surface to capture the strain in one direction, based on the change of electrical resistance, due to the change of length and diminution of transverse section of the wire by the Poisson effect. 

The gauge factor (GF), defined as the ratio of relative change of electrical resistance to strain, is given by GF = (ΔR/R_0_)/ε. The GF is usually around 2, slightly higher than 1 + 2ν, because of the weak piezoresistive behavior of Constantan (Cu–Ni alloy), the metallic alloy used for conventional strain gauges. Semiconductor strain gauges made with Si and Ge have a GF of about 100, because of their strong piezoresistive behavior [1].

The metal wire gets the shape of a grid to minimize its size. The main quality of a good strain gauge is that the transverse sensitivity, or the response to strains not oriented with the wire direction, is kept as low as possible—usually only few percent. This fact allows rosettes, combinations of three strain gauges, to be built. These are used to get the three components of the strains in a plane.

Another procedure used to get the strain components experimentally is Digital Image Correlation (DIC) [2], which captures the in-plane displacements of the surface points in a small area, producing a map of the strain field and the Fiber Bragg gratings [3], among other procedures. After 1998 [4], the dispersion of a low percentage of carbon nanotubes (CNTs) in polymers, and even on concrete, was attempted, and piezoresistive behavior was demonstrated. Compared with existing technologies, CNTs offer some unique advantages as sensors. They can be easily adapted to complex surface geometries; they can either be built-in and embedded into a material or built onto a substrate film, and then bonded to the surface in a conventional way. If the dispersion is done on an elastomeric polymer, the development of sensors with a very high strain capability—over 600%—is feasible. These polymers are referred to as highly stretchable sensitive materials or sensitive skins, and they are very useful for biomedical and biomechanics applications.

The electrical conductivity of polymers greatly increases with the addition of CNTs. Hu et al. [5] did a review of existing results and proposed a statistical model to predict the response of the sensor under tensile and compressive stresses and the influences of the type of CNT and polymer, the weight fraction, and the processing parameters. Experimental results and numerical predictions showed that conductivity change from 0.1 S/m, for an MWCNT weight percentage of 0.5%, to 10 S/m (1%). Using numerical simulation and experimental verification, it was also found that electrical resistance increases with increasing strains, following a linear-exponential law, due to the electrical tunneling transport effect. Furthermore, changes in compression were shown to be much smaller than those for tensile loads, and the relative changes were shown to be higher for smaller CNT concentrations. When blended with epoxy resin, even the cure temperature was found to have an influence on the gauge factor, but the diameters of the nanotubes and their conductivity play more dominant roles, together with the nature of the polymer matrix. In this regard, Wagner et al. [6] also reviewed the parameters influencing the Gauge Factor (GF). 

**Figure 1:**
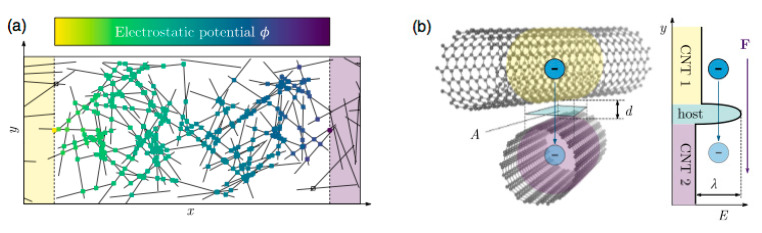
Sketch of electrical conduction in randomly dispersed carbon nanotubes (CNTs) in doped resins. (**a**) Randomly oriented CNT. (**b**) Contact resistance among nanotubes (from reference [6], with CC permission).

The electrical resistivity of a randomly dispersed CNT–polymer composite can be attributed to two main factors, as sketched in Figure 1 (taken from reference [6]): the intrinsic electrical resistance of the nanotubes, and the contact resistance among adjacent nanotubes. Since the gap between CNT contact points is filled with polymers, the contact resistance or tunneling resistance is several orders of magnitude higher than the inherent carbon nanotube resistance. It is worth noting that the electrical flow will be mainly quasi-isotropic, independent of the locations of the electrodes. The distance among contact points increases by tensile strains, thereby increasing the tunneling resistance.

It is worth clarifying that this paper only deals with non-oriented CNT sensors working at low levels of strain, which are the most common case, and are typically used in structural applications. A few papers describe the production and characterization of sensors with aligned CNTs, which exhibit quite a different phenomenology, with a clear anisotropy along the CNT direction [7,8]. Moreover, there are also a large number of articles dealing with CNTs dispersed in elastomers, which allow for very high levels of elastic strain—over 100%. The load–unload cycles of these sensors cause a reorientation of the CNTs, and some singular phenomena, like an increase in the GF and hysteresis effects, occur. A discussion and model of the behavior of these highly stretchable sensors can be found in [9]. For structural applications, the limit of 1%–3% for elastic strain is more than enough. Metallic alloys used to have an elastic limit below 1%, depending on the quenching state, and similar limit applies for CFRP (Carbon Fiber Reinforced Plastics). 

To get the highest sensor sensitivity, it is clear that the CNT concentration should be kept as low as possible, only slightly over the “percolation threshold”, defined as the critical volume fraction when the nanocomposite changes from an insulator to a conductor, through the establishment of a conductive network inside the insulating matrix. Bauhofer et al. [10] reviewed the influences of different matrices and dispersion procedures. It is worth mentioning that epoxy resins have smaller percolation values, in some cases even below 0.1 %wt., and gauge factors close to 25 have been reported. 

It has been found that the strain sensitivity of CNT/polymer composite sensors is better than that of pure CNT sensors, like buckypapers [11]. The type of polymer is one of the main influential factors, but other parameters that may influence the strain sensitivity are the fabrication technique used, the type of surfactant and dispersion technique used, the quality and quantity of CNTs used, the alignment and entanglement of CNTs, and the environmental conditions. Similar conclusions were made by Kanoun et al. [12], who reviewed over 125 articles with a variety of processing routes. One of the main conclusions was that CNT agglomerates cause a loss of repeatability. For this reason, it is very important to establish adequate dispersion techniques, in order to fabricate good strain sensors.

Two basic approaches are used to produce the sensor films. One way is to grow CNTs on an adequate support, like glass fibers, by CVD (Chemical Vapor Deposition), and then embed these fibers inside the structural material [13]. The other approach, which is more commonly used, because of its simplicity, consists of preparing a homogeneous dispersion of CNT with adequate surfactant, and then transferring the solution to a resin film by drop casting, inkjet printing, or spray coating. A detailed description of the steps done to produce good sensors can be found in two doctoral thesis reports [14] and [15]. L. Jin et al. [9] produced sensing film after several passes with spray coating by controlling the resistance value among the electrodes. This seems to be a good way to achieve greater uniformity and repeatability.

### Transverse Sensitivity: Review of the Results Reported in the Literature

**Figure 2:**
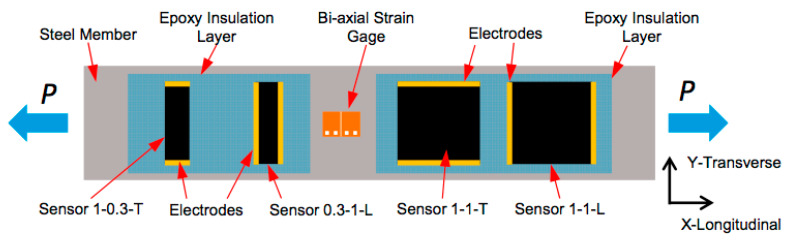
Uniaxial tensile test of a steel plate with longitudinal and transverse sensors. (from reference [16], with permission).

**Figure 3:**
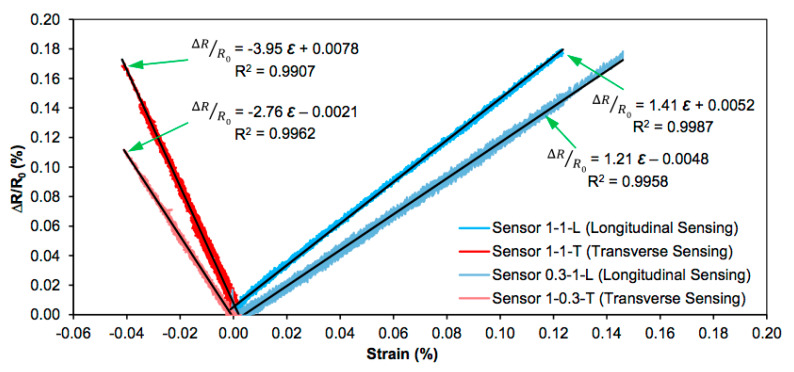
Experimental results obtained from the former specimen. The abscissa axis shows the longitudinal or transverse strain measured experimentally with a biaxial strain gauge (from reference [16], with permission from CC4).

Characterization of the mechanical response has mainly been done by uniaxial tests. Figure 2 and Figure 3 are taken from one of the earliest works [16] that tried to explore transverse sensitivity, assuming that a sensor is transversally oriented if the electrode line is perpendicular to the applied load. They obtained negative gauge factors for these transverse sensors, meaning that the resistance also increased when related to the transverse strain, which was negative. All sensors bonded to the same metallic plate were obviously submitted to the same levels of longitudinal and transverse strains. 

Using the steel properties (E = 200 GPa, ν = 0.3) it is easy to calculate that the maximum uniaxial longitudinal stress applied to the former test was 240 MPa, which corresponded to longitudinal and transverse strain levels of +0.0012 and –0.0004, respectively. The maximal relative electrical resistance change given in the former figure is approximately 0.0017 for three cases and 0.0012 for the 1-0.3-T sensor. The responses of the longitudinal 1-1-L and transverse sensor 1-1-T were quite similar, irrespective of the electrode positions. The size of the sensors was shown to be more relevant for the electrical response than the position of the electrodes, as can be seen by comparing the 1-1-T and 1-0.3-T sensors.

The former paper was the first, but not the only one, to report this “anomaly”. Negative gauge factors with transverse strains have also been reported by other authors [17,18]. Under a uniaxial tensile load, the resistance always increases, no matter where the electrodes are put. Reference [18] concluded “the largest deformation dictates the change in electrical resistance in all directions.” In reference [19], an interesting experiment is described. A CNT buckypaper, the size of a 30 mm diameter disk, was bonded to a glass/epoxy plate, and then submitted to a uniaxial load. Several electrode wires were bonded at the perimeter of the circle, and electrical resistance changes were monitored for pairs of electrodes defining diameters at 0°, 30°, 60°, and 90°. In this way, the distance between electrodes was always the same. All of them gave a nearly identical response, demonstrating, once again, that the electrical response is uncorrelated with the relative directions of the strains and electrode line.

One of the first papers to use this kind of sensor under biaxial loads was [20], in which a pressure vessel was monitored. Conventional and CNT sensors were put in the longitudinal and circumferential directions, but the comparison of experimental measurements produced rather inconclusive results, and the results obtained with the CNT sensors showed poor reproducibility under cyclic loads, and did not gave very useful information. An explanation of how to use the “negative gauge factor” was not given, so data analysis could not be properly done. A few papers have claimed the capability of determining the direction of principal strains [21], but, as they did not even consider the tensorial nature of the strains, their approaches must be considered with caution. 

Recently, an approach that would allow the identification of the three components of the in-plane strain tensor was proposed [22]. By assuming that the resistivity of CNT film becomes anisotropic as a consequence of strains, the following relationship, similar to the existing one among strain–stress tensors, was postulated:

(1) $$\frac{1}{\rho }\left\{\begin{array}{c} \Delta \rho_{x}\\ \Delta \rho_{y}\\ \Delta \rho_{z} \end{array}\right\}=\left(\begin{array}{c} m_{11}\\ \begin{array}{c} m_{21}\\ 0 \end{array} \end{array}\begin{array}{c} m_{12}\\ \begin{array}{c} m_{22}\\ 0 \end{array} \end{array}\begin{array}{c} 0\\ \begin{array}{c} 0\\ m_{33} \end{array} \end{array}\right)\;\;\left\{\begin{array}{c} \varepsilon_{xx}\\ \varepsilon_{yy}\\ \varepsilon_{xy} \end{array}\right\}$$

In order to validate this hypothesis, sensors with at least four electrodes are needed to measure the resistivity changes in at least two directions. A group from Keppler University (Austria) is doing interesting works on EIT (Electrical Impedance Tomography) [23] using this kind of multielectrode sensor for damage detection in graphite/epoxy laminates, which have a strong level of anisotropic resistivity. The same authors recently published a paper detailing the application of this method to strain measurement in a single lap bonded joint [24], where some practical difficulties were identified due to the small changes in resistivity of the CNT-doped films and the inherent low resolution of the EIT technique.

Our experiments, described in detail in the next section, confirm that transverse sensors have a positive resistance change, but also asked two more relevant questions:

Do the positions of the electrodes define the direction of the strain that is being measured?
Is it possible to obtain the strain components individually, as suggested by [22]?

## 2. Materials and Methods

The initial goal for this work was simply to compare the strain sensing capabilities of fiber optic and CNT sensors in a non-uniform strain-state condition, and to learn about the response of CNT sensors to high shear strain. A double lap bonded joint was proposed, because such joints are known to have strong strain gradients. Compared with the more common single lap bonded joint, double lap joints avoid the bending moments caused by load eccentricity; bending moments add flexural strains and make it more difficult to properly understand the experimental results. 

When reducing the obtained data, we encountered the much more important issue of the sensor directionality.

### 2.1. Double Lap CFRP Bonded Joint

A previously published paper [25] details the adequate conditions to prepare a CNT-doped adhesive, as well as the resulting mechanical and electrical properties in a standardized single lap joint. The MWCNTs used for this study were NC7000, which were supplied by Nanocyl. They had an average diameter of 10 nm and a length of up to 2 μm. The procedure consisted of preparing an aqueous dispersion of CNTs with a sodium dodecyl sulfate (SDS) surfactant by means of ultrasonication, which was then sprayed over a high quality commercial uncured epoxy adhesive film, FM300K. It was found that if done adequately, it did not significantly influence the mechanical properties, and the desired values of electrical conductivity in the plane and through the thickness were achieved, suggesting that CNTs penetrate through the resin during curing. In this study, the optimum weight fractions of CNTs and SDS were set as 0.1 wt.% and 0.25 wt.% to maximize the electrical and mechanical behavior of the joints. It is worth mentioning that, for manufacturing procedures based on aqueous dispersions of CNTs, like ink coating or spraying, the final percentage CNT/resin is usually unknown. It can be determined by weighting the resin film before spraying and after evaporation of the solvent, which is not usually done. The right percentage is established by successive spray passes, until adequate electrical and sensing properties are achieved. 

**Figure 4:**
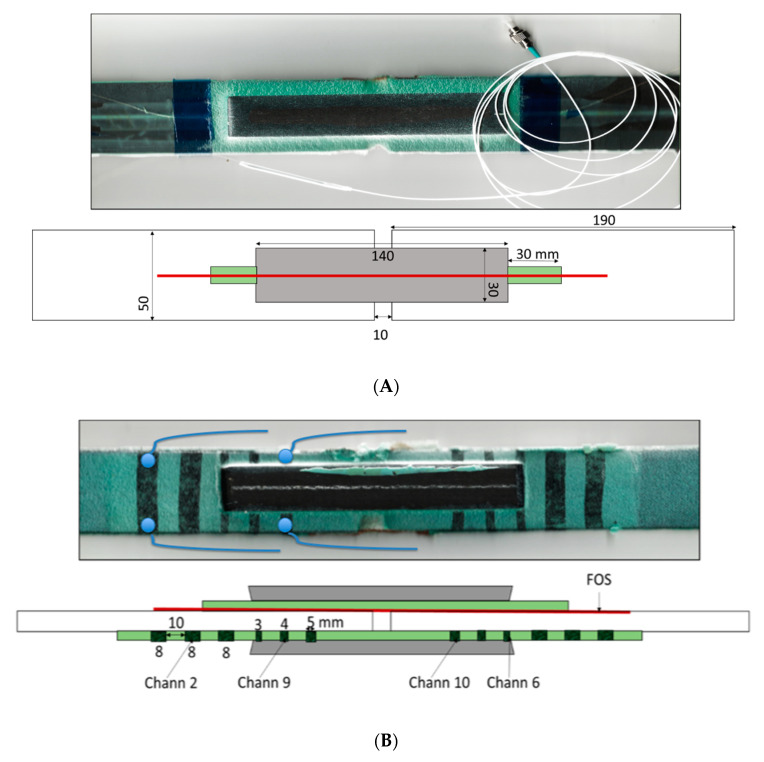
(**A**) Double lap bonded joint specimen.Photograph of one side of the specimen, constructed with a longitudinally aligned optical fiber. (**B**) Double lap bonded joint specimen. Photograph of the other side of the specimen, where the transverse black strips are CNT-doped regions on the green adhesive. Copper wires were used as electrodes, fixed with silver conductive paint, and sealed with an adhesive layer to avoid environmental issues, as sketched in the figure.

The dimensions of the specimens are sketched at Figure 4, with pictures from the two sides of the specimens. One side of the specimen includes conductive strips with doped adhesive film, while the other side has one embedded optical fiber, which was included with the adhesive film before curing.

Adherents were done with CFRP laminates made with 13 plies of CYCOM 977-2 unidirectional tape with a layup of [(0,90)_3_,0]s. The basic properties of the laminae and the laminate, which were obtained by CLT (Classical lamination ´theory) using ESACOMP software, are given in Table 1 and Table 2, respectively.

CYCOM 977-2 is an advanced resin system that comes with intermediate modulus carbon fibers to be processed in an autoclave, but it is still able to produce good quality laminates under Out of Autoclave (OOA) conditions, as were used for this work. 

A co-bonded process was undertaken. Firstly, central laminates were cured and cut to the requested size. Then, the optical fiber was located at a small groove on the surface to fix its position and to avoid any relative movement. 

An uncured adhesive film, FM300K, with a thickness of 0.22 mm and a shear modulus G of 0.907 GPa, was added onto it. Then, an uncured laminate with the size of the upper adherent was added on top, and finally, a vacuum bag was made and the assembly was cured in an oven with the prescribed cure cycle, that is, at a temperature of 180 °C for 2 h. 

As a second step, the assembly was turned upside-down and, again, a fresh adhesive layer was added onto it, and the ink with CNT was spread at the defined positions sketched in Figure 4. It was found that the adhesive has a significant flow under the curing conditions. Micrographs taken in previous works [26] demonstrated that the carbon nanotubes were well dispersed with a random pattern. In order to get a higher sensitivity (GF) linked to the final percentage of CNT in the cured resin—the optimal being near the percolation threshold—it was decided to cure the first layer of adhesive, and then add a second adhesive layer and the uncured laminate for the upper adherent. It is important to mention that the width of this laminate was slightly smaller than that of the central laminate, to make the installation of electrodes for the CNT strips easier, as can be seen at the pictures below.

### 2.2. Uniaxial Tensile Load on Aluminum Specimens with a Bonded CNT Sensor

**Figure 5:**
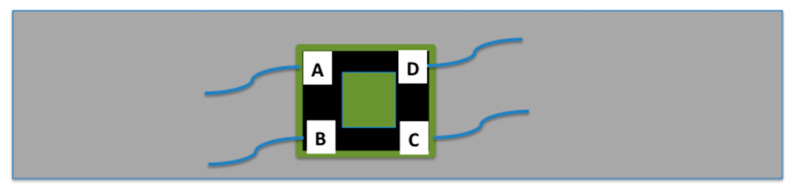
Uniaxial tensile specimens made with aluminum with a CNT bonded sensor, and electrodes at the corners (size of the sensor 20 × 20 mm). The black strips are the doped areas, 5 mm in width.

As a result of the striking results obtained by the CNT sensors during the former experiment (results discussed latter in this paper in Section 3), we decided to reproduce the experiments proposed in reference [22] to get a better understanding of the directionality of the response. A sensor constructed as a square 20 mm × 20 mm doped uncured adhesive, with the same characteristics as described in Section 2.1, was put onto an aluminum plate. Wire electrodes were put at the four corners with a point contact within the doped layer. A schematic of the electrode disposition is shown in Figure 5. Here, electrodes are marked A, B, C, and D. 

In the experiment proposed in [22], the doped area covered the whole surface of the square and pin electrodes were used. Without load, the electrical resistance among the four corners should be similar, but we measured significant differences, up to 50%, either because of poor contact points or because of inhomogeneities and agglomerates in the CNT distribution (sprayed in several passes over the fresh adhesive). As a result of this, to get a better reproducibility, we built the sensor as depicted at Figure 5. The black strips represent the doped area over the fresh adhesive, 5 mm wide, and a large dot of silver paste was used at the corners to guarantee a good electrical contact. 

Strain gauges were not included on the aluminum plate, as longitudinal and transverse strains can be easily derived from the uniaxial stresses (elastic properties for aluminum used at this work: E = 70 GPa, ν = 0.3).

## 3. Experimental Results

### 3.1. Test Results from Distributed Fiber Optic Sensors

Distributed strain measurements were obtained by an OBR Luna 4600 (Luna Inc., Roanoke, VA, USA), one of the most advanced system in the fiber optic sensing market, offering a spatial resolution as good as 0.3 mm. Distributed sensing by OBR has been one of the most significant advances on fiber optics sensors in the last two decades. It is based on the Rayleigh dispersion of light from every point of the optical fiber (OF), occurring due to slight inhomogeneities randomly distributed inside the core of the OF. Each optical fiber has a unique reflectivity pattern (attenuation.VS. length). By Optical Frequency Domain Reflectometry (OFDR), the signal from an fiber submitted to strain-temperature changes is compared to the initial reference signal, and, after a cross-correlation analysis, local strain/temperature changes can be calculated by means of a spectral shift conversion. More details on the physical principles can be found in [3].

From a user point of view, it is important to choose a right balance among spatial resolution and strain accuracy, both terms influencing in opposite way. Getting very high spatial resolution means to shorten the ‘sensor length’, diminishing the number of data points taken to calculate the spectral shift, then adding noise to the strain measurements. For our tests, because we were expecting strong strain gradients, we found the optimal value to be 5 mm, getting a smooth strain line. 

**Figure 6:**
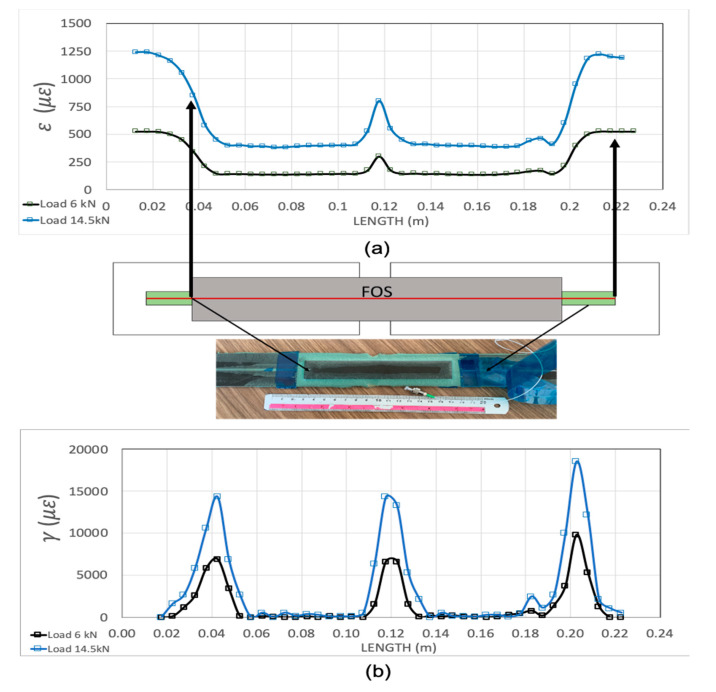
Experimental axial strain (**a**) and shear strain (**b**) at the double lap joint, obtained by distributed optical fiber sensing.

Figure 6 gives the axial strain field obtained by the embedded optical fiber attached to the central laminate. The response was linear with applied loads up to the load level tests carried out.

As mentioned above, a plain, optical, acrylate coated fiber was located in a small groove made at the surface of the central laminate before the adhesive was cured, so it received the axial strain of the laminate. In fact, for the region outside the bonded joint, the measured strain for an applied load of 14.5 kN was 1250 με, which was in good agreement with the numerical predictions for this laminate. ESACOMP software, using the conventional Classical Lamination Theory (CLT), predicted an axial strain ε_xx_ of 1245 με for an applied load of 300 kN/m (15 kN for a specimen width of 50 mm), quite similar to that obtained experimentally. At the overlap region, the axial strain dropped by about one third because of the higher stiffness of the specimen at this region. 

Shear strains cannot be directly measured by this fiber optic technique, but according to the Volkorsen model, the spatial derivative of this axial strain is proportional to the shear strain field multiplied by the stiffness ratio E_adherent_/G_adhesive_. The derivative of experimental axial strain is also included in Figure 6 (obtained as Δε/Δx), showing the classical “bathtub” shape. Shear strain peaks at both ends of the overlapped regions. The acrylate coating of the optical fiber introduced a slight “shear lag” into the measurements, and, because of it, the drop at both ends is not vertical, as it should be. 

### 3.2. Test Results for the Adhesive Joint from CNT-Doped Films

**Figure 7:**
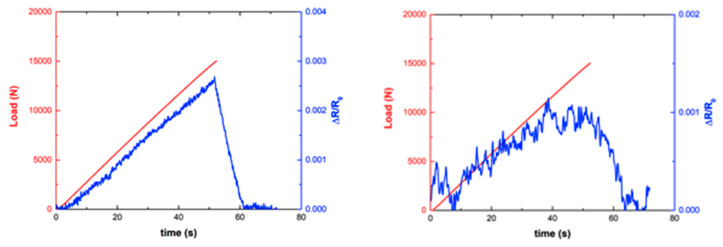
The electrical response as a function of the mechanical load for channels 2 (**left**) and 10 (**right**). Channel 2 was outside the bonded area, while channel 10 was inside the bonded region (see Figure 4).

**Figure 8:**
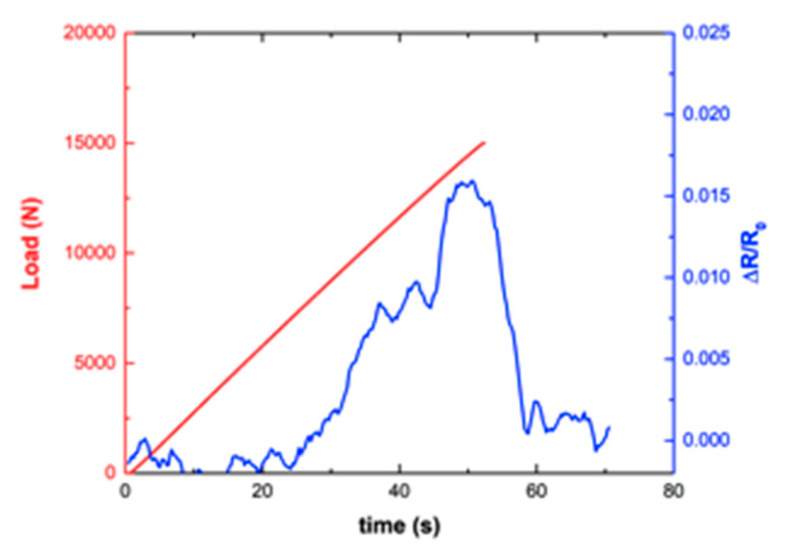
Electrical response as a function of the mechanical load for Channel 6.

The electrical resistance of the doped channels was determined during the mechanical tests by an Agilent 34970A at an acquisition frequency of 10 Hz. Some of the obtained results are presented in Figure 7 and Figure 8.

The first important conclusion is that the direction of the conductive strip does not limit the readings. In our case, the strips were transversally located, but the readings were mainly longitudinal strain, which was responsible for a change in distance among the nanotubes. 

Tests were done under load control of the MTS test machine at a rate of 300 N/s up to 15 kN below the failure load, so re-testing was possible. It is easy to calculate that for the maximum load, 15 kN, there was an longitudinal stress of 122.75 MPa (for the basic laminate, outside the bonded region, the transverse section was 2.444 × 50 mm^2^), and the longitudinal and transverse strain were +1245 and −37.5 µε, respectively (the cross-ply laminate has E_xx_ = 98.60 GPa, a near zero equivalent Poisson ratio, as given at Table 2). Inside the bonded region, where three laminates were put together, the strain level was approximately three times lower. Additionally, it is worth mentioning the linearity of the electrical response at channel 2. For the maximal load, at a strain level of 0.001245, we got a relative electrical change ΔR/R_0_ of 0.0026, giving a gauge factor of slightly over 2. 

The response of channel 10, well inside the bonded region (Figure 8) was similar to the former one, but possibly noisier because of the smaller width of the sensor. For the maximum load, the relative change in resistance was ΔR/R_0_ = 0.001, about one-third that of channel 2, which is explained by the lower level of strain in this area, as explained previously. 

The response of channel 6, located in the high shear strain region, was quite different, not only because of the loss of linearity, but also because of the much higher values of resistance change, one order of magnitude higher than those of channel 2, with some permanent resistance changes, suggesting the occurrence of microcracking and permanent damage or possibly permanent reorientation of CNTs.

In this context, it can be observed that channel 6 (inside the bonded area, at the end of the overlap) showed the highest variation in electrical resistance with the applied load (around 1.5%), whereas channel 10 (inside the bonded area, in the middle region) showed the least amount of electrical change during the tests (around 0.1%). Finally, channel 2 (outside the bonded area) showed a good linear response with intermediate variation between channels 6 and 10 (around 0.3%).

We suspect that the high shear strain (over 10,000 με) experienced by resin at channel 6 is responsible for the large increase of its electrical resistance. However, we cannot forget that this region is also subjected to high peeling stresses and, consequently, a high level of normal strain is also present, as can be predicted by the more advanced Goland–Reisssner theory, or by a detailed Finite Element Model (FEM). The main conclusion is that quantitative values of the strain components cannot be determined using this technique, because three components cannot be determined using only one scalar measurement. More advanced techniques like DIC [2] should be used.

### 3.3. Test Results for the Uniaxial Loaded Aluminium Plates with CNT Sensors 

For the aluminum tensile specimens with a solid square sensor, when submitted to uniaxial tensile load, a noisy response with unexpected irregularities occurred. To simplify and clarify this presentation, the values of the initial electrical resistance and the relative resistance change for each pair of electrodes at a stress level of 70 MPa are given at Table 3. A uniaxial stress of 70 MPa corresponds to an axial strain of 0.001 (0.1%) and a transversal strain of –0.0003 (–0.03%), based on the values for aluminum alloys: E = 70 GPa and ν = 0.3). 

A disperse response was found for the equivalent paths AB and CD of the square sensor. They had quite different initial levels of resistance, with differences higher than 50%, and similar comments can be made concerning the other results. The gauge factor was not the same for any of the paths. We concluded that this experiment, as proposed at reference [22], is difficult to execute (in reference [22], a difference of only 5% was mentioned). Our initial results had a low level of reliability, making it difficult to draw any conclusions from them. 

**Figure 9:**
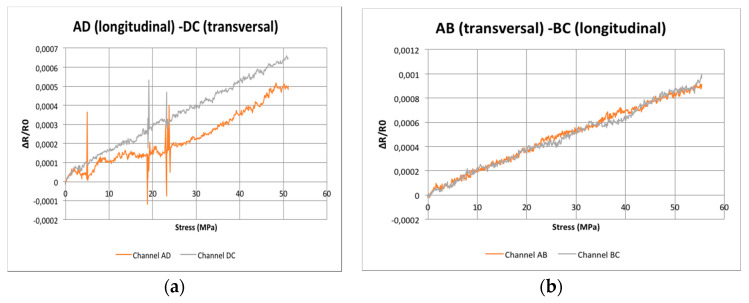
Longitudinal and transversal responses of carbon nanotube (CNT) sensors bonded to an aluminum plate and submitted to uniaxial loads. (**a**) Resistance changes for AD and DC paths. (**b**) Resitance changes for AB and BC paths.

Our measurements improved when the doped area only covered the sides of the square, as depicted in Figure 5, and when the electrodes had an additional dot of silver paste to ensure good electrical contact. In fact, the initial value of the electrical resistance was around 6600 Ω, regardless of the channel, which indicates better electrical contact and dispersion of the CNTs in the stripes. The results are presented at Figure 9, where the abscissa axis represents the uniaxial longitudinal stress, which produces longitudinal strain (ε_xx_ = σ_xx_/E) and negative transverse strain (ε_yy_ = −ν σ_xx_/E).

It is remarkable that, in the second figure, the responses of longitudinal or transversely oriented sensors are quite similar. However, there are still some differences when comparing AB to DC.

It is true that the sensitivity achieved by the developed sensors is not high. In this regard, it could be increased by adding, for example, a centrifugation step after ultrasonication process, in order to achieve a better CNT dispersion, as well as to increase the resistivity, leading to higher sensitivities because of a higher interparticle distance. Another way would be the chemical functionalization of the CNTs, which has been proven as an effective method to increase the sensitivity [27].

## 4. Discussion

The response to strain from polymer films with randomly dispersed CNTs is not similar to that experienced by electrical strain gauges, because there is a coupled response to both longitudinal and transverse strains, as has been clearly demonstrated by this and previously published works, even though its consequences had not been clearly stated before. It is accepted (without any supporting experimental evidence) that the positions of the electrodes define the current flow, and that the electrical resistance changes are due to strains applied in this direction. Our experiment on the double lap bonded joint demonstrated a linear response among the axial stress, and a change in resistance for a sensor attached to the laminate outside the bonded region with transversal electrodes. The laminate was made with a cross ply layup, with a near zero Poisson ratio and, consequently, had quite low transversal strain. Under tensile load, the resistance change was always positive, even though the transversal strain was negative.

These results suggest that the definition of the gauge factor as the ratio among relative resistance changes and strain in one direction is valid for conventional strain gauges, because these artifacts are built to avoid transversal sensitivity. However, this definition is no longer valid for CNT doped films; it may work for uniaxial tests, but will fail for a more general biaxial stress state. 

Our first question can be answered without doubt: the positions of the electrodes do not define the direction in which the strains are measured. In fact, the electrical response to an external load is nearly independent of the electrode positions; their influence is more closely related to the distances among electrodes and electrical contacts. More specifically, good electrical contacts are needed to get reliable measurements. Here, a high contact resistance is parasitic and degrades the sensor’s performance.

Regarding the second question, whether it is feasible to determine the different components of the strain tensor, it is clear that, with just one measurement, as is obtained with two electrodes, three independent variables cannot be determined. This obvious statement has important consequences, for example, in the case of biaxial loads, like a pressure vessel, as mentioned at the Introduction, the axial and hoop strain components cannot be obtained by a CNT sensor with two electrodes, no matter where these electrodes are put. This introduces two additional questions:

(a) Can multi-electrodes sensors distinguish among strain components, as proposed in [22], by measuring the electrical anisotropy caused by strains?
(b) For the conventional two electrode sensors, what is the relationship among the measured electrical resistance changes and the strain state?

Concerning question (a), we tried unsuccessfully to produce the four electrode arrangement with adequate reproducibility, but we were able to measure resistivity in two orthogonal directions. Our results, similar to other published results [16], indicate that the resistance changes with strain are nearly the same in both directions, within the limits of the accuracy of the measurement system. The main issue for the model proposed in reference [22] is that the electrical anisotropy caused by the strains is always very small, about 0.1%. So, it would be faded out by the local inhomogeneities of the CNT distribution and agglomerates, which cause, under the best conditions, resistivity differences higher than 5% from sensor to sensor and inside the same sensor, if measured in two orthogonal directions. Consequently, strain components cannot be determined by this method.

Concerning question (b), our hypothesis is that resistance changes are related to a term that is the sum of the components of the main diagonal of the strain tensor, also known as the “first invariant”, because this sum will not change with a change in the coordinate reference system, or “volumetric strain”, because it reflects the change in volume of the solid, while the other components are called “deviatoric”, because they change the shape but not the volume. According to the theories for electrical conductivity of CNT–resin composites [28], the changes in resistivity are related to changes in distance among adjacent nanotubes (Figure 1), and these statistically distributed changes of distance seem to be related to the global change in volume. To validate this hypothesis, biaxial tests are needed, which requires special cross-shape specimens and biaxial test machines that are not commonly available [29]. We will try to validate it in the mid-term future.

This behavior means a severe limitation for the possibilities of CNT-doped films as strain sensors when compared to alternatives, such as strain gauges or fiber optic sensors, which are able to distinguish the strain components, but this is similar to the behavior of piezoelectric films, whose response is also insensitive to the direction of in-plane strains. In an excellent review on graphene-based strain sensors [30], the similarities among resistive, capacitive, and piezoelectric sensors were also highlighted. 

Some authors [31] have explored this similarity, demonstrating that sensors made from carbon black/Polyvinylpyrrolidone (CB/PVP) nanocomposite ink also work under the principle of conductivity, based on tunneling current among conductive particles. They demonstrated that these sensors, working at frequencies as high as 400 kHz, are able to detect the subtle strain changes associated with elastic waves, a task normally reserved for piezoelectric wafers. 

## 5. Conclusions

This paper only deals with non-oriented CNT sensors working at low levels of strains, which are the most common case, and are typically used in structural applications. We have tried to answer three basic questions:

1. Do the positions of the electrodes define the strain direction that is being measured?
2. Is it possible to obtain the individual strain components using CNT sensors?
3. What is the relationship between the measured electrical resistance changes and the strain state?

For the first two questions, the answers were found to be negative. The electrical response of a piezoresistive CNT sensor is always the same, regardless of where the electrodes are put or their relative positions to the applied loads. For the second question, it is obvious, but frequently forgotten, that with only one measurement (change in electrical resistance), information about three independent variables cannot be obtained. Even for a uniaxial load on an anisotropic specimen, the transverse strains cannot be experimentally obtained with piezoresistive sensors. 

For the last question, we propose that resistivity changes are related to the sum of the principal strains, the diagonal term of the strain tensor. This is in full agreement with the former two results, and there is scientific support that this term is related to the change in volume of the material submitted to strains. However, this theory needs to be validated by biaxial tests, which are not easily available.

## Figures and Tables

**Table 1:** Material properties of CYCOM 977-2.

Moduli (GPa)		Poisson’s Ratios	
E_11_	175.00	ν_12_	0.30
E_22_	8.68		
G_12_	4.30		

**Table 2:** Laminate stiffness properties. (Calculated by ESACOMP).

Laminate: Lay-up:((0/90)_3_/0)SO h = 2.444 mm			
In plane Modulus (GPa)			
E_xx_	98.60	ν_xy_	0.0305
E_yy_	85.76		
G_xy_	4.30		

**Table 3:** Results from a solid square doped adhesive bonded to aluminum.

	AB	CD	AD	BC	BD	AC
	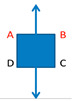	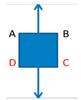	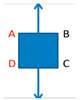	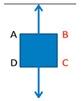	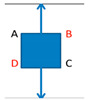	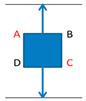
Initial resistance (ohms)	32,356.761	48,379.295	42,509.651	35,019.153	44,062.44	38,965.256
(∆R/R_0_) for σ = 70 MPa	0.000288	0.002433	0.000940	0.001320	0.001610	0.000758
